# Positive effects of neuromuscular exercises on pain and active range of motion in idiopathic frozen shoulder: a randomized controlled trial

**DOI:** 10.1186/s12891-023-06173-8

**Published:** 2023-01-20

**Authors:** Lu Wang, Ge Yu, Ran Zhang, Guangyan Wu, Lei He, Yaping Chen

**Affiliations:** Department of Rehabilitation, Beijing Tongren Hospital, Capital Medical University, 1 Dongjiaominxiang, Beijing, 100730 China

**Keywords:** Neuromuscular exercise, Maitland technique, Idiopathic frozen shoulder, Regular physical therapy, Rehabilitation

## Abstract

**Background and objectives:**

Frozen shoulder (FS) is characterized by pain and significant loss of active and passive shoulder motion. Strengthening exercises are among the standard exercises used for FS. Neuromuscular exercise (NME) effectively improved pain and the range of motion in shoulder. However, no prior research has looked into the effects of NME compared to strengthening exercises in FS rehabilitation. The aim of the present study was to evaluate the effects of NME compared to strengthening exercises on pain and active range of motion (AROM) in individuals with idiopathic frozen shoulder.

**Methods:**

Forty individuals with idiopathic frozen shoulder were randomly assigned to either the experimental group (NME with regular physical therapy, *n* = 20) or the control group (strengthening exercises with regular physical therapy, *n* = 20). In both groups, the interventions were performed once a day, 5 days a week for 8 weeks. Pain scores on the visual analogue scale (VAS) and AROM of the shoulder were assessed at baseline and after the 8-week treatment. The primary analysis was the group × time interaction.

**Results:**

Two-by-two mixed analysis of variance (ANOVA) revealed a significant group × time interaction for VAS (F = 29.67; *p* < 0.01); AROM in flexion (F = 12.05; *p* < 0.01), internal rotation (F = 6.62; *p* < 0.05) and external rotation (F = 16.93; *p* < 0.01) in favor of the experimental group. The two-by-two mixed ANOVA revealed a significant main effect of time for VAS (F = 1648.47; *p* < 0.01); AROM in flexion (F = 591.70; *p* < 0.01), extension (F = 114.57; *p* < 0.01), abduction (F = 1602.04; *p* < 0.01), internal rotation (F = 664.14; *p* < 0.01) and external rotation (F = 1096.92; *p* < 0.01). No other significant differences were found.

**Conclusions:**

NME is superior to strengthening exercises in terms of pain and AROM of shoulder flexion, internal rotation and external rotation in individuals with idiopathic FS. NME could be used to treat individuals with FS.

**Trial registration:**

Trial registration number: ChiCTR2100054453. Registration date: 17/12/2021.

**Supplementary Information:**

The online version contains supplementary material available at 10.1186/s12891-023-06173-8.

## Introduction

Frozen shoulder (FS) or adhesive capsulitis is an unknown intrinsic disease associated with spontaneously progressive inflammation and fibrosis of the shoulder joint capsule, characterised by pain and significant loss of active and passive shoulder motion [1]. Frozen shoulder (FS) or adhesive capsulitis is an unknown intrinsic disease associated with spontaneously progressive inflammation and fibrosis of the shoulder joint capsule, characterised by pain and significant loss of active and passive shoulder motion [2]. The prevalence rate is 2–5%, affecting more women than men aged 40–60 years, and the incidence rate increases with age [3–5]. The clinical presentation of FS is described in three overlapping stages. The freezing stage is associated with pain that is typically worst at night and can last from weeks to 9 months. The frozen stage is characterized by progressive loss of range of motion and marked stiffness with gradual reduction in pain and can last from 9 to 15 months. In the thawing stage, the pain and stiffness gradually subside, but significant stiffness persists for 15 to 24 months [6, 7]. Pain and limitation of movement are the most common complaints affecting shoulder function in activities of daily living and quality of life [4, 8, 9]. Apart from surgery, conservative treatments are common for FS and include individual education, oral or intra-articular glucocorticoids, physiotherapy and exercise programmes [10–12]. Strengthening exercises are among the standard exercises used for FS [13–15].

Sensory and proprioceptive input may be reduced if the shoulder cannot be moved due to pain in FS [16, 17]. And proprioception plays an important role in sensorimotor control, especially in the shoulder complex, which depends significantly on joint stability during movement [18]. Stabilisation must be offered to allow smooth movement of the distal joints. Neuromuscular control is crucial for maintaining dynamic stabilisation [19]. Neuromuscular exercise (NME) involves motor control, (re) learning and proprioceptive training that addresses the quality of movement and emphasises joint control [20]. It is increasingly used to facilitate faster recovery in individuals with shoulder dysfunction with shoulder pain and impaired proprioceptive function such as traumatic anterior shoulder dislocation [21, 22], It is increasingly used to facilitate faster recovery in individuals with shoulder dysfunction with shoulder pain and impaired proprioceptive function such as traumatic anterior shoulder dislocation [23] and rotator cuff tendinopathy [24].

As for comparing the effects of NME and strengthening exercises for the shoulder, Eshoj et al. [21] showed that NME improved shoulder pain and function more than strengthening exercises in individuals with traumatic anterior dislocation. Gin et al. [25] showed that NME is as effective as strengthening exercises in treating chronic shoulder pain. Ageberg et al. [26] showed that NME was as effective as strengthening exercises in degenerative knee disease. Risberg et al. [27] demonstrated that NME significantly improved pain and knee function compared to strengthening exercises at the 6-month follow-up after anterior cruciate ligament reconstruction. As far as we know, no study has looked at the effects of NME compared to strengthening exercises in the rehabilitation of FS.

Therefore, the aim of the current study was to compare the effects of NME and strengthening exercises at FS. We hypothesised that NME may improve the symptoms of FS better than strengthening exercises.

## Materials and methods

### Study design

**Fig. 1:**
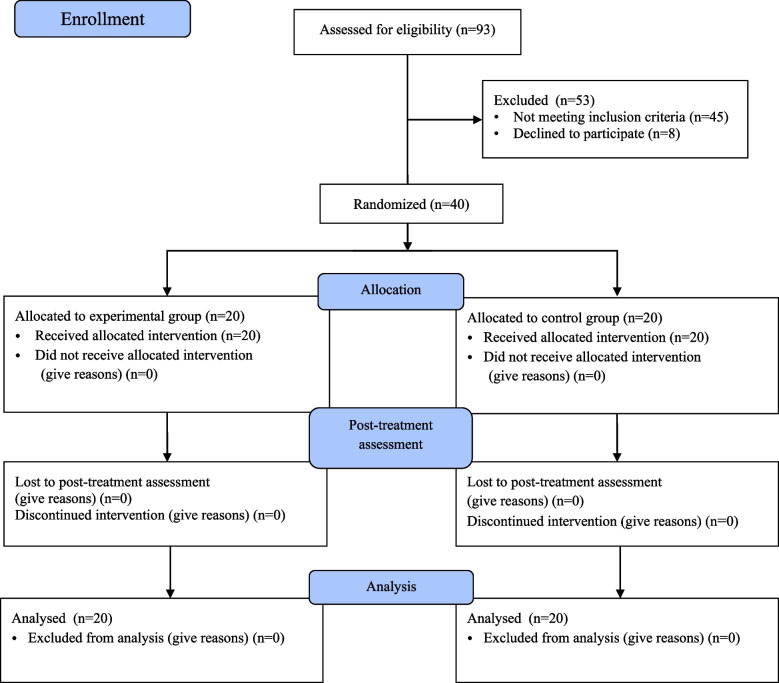
CONSORT flow diagram

This was a single-blinded, randomised controlled trial conducted in the rehabilitation medicine department of a regional hospital. Forty participants were recruited from December 2021 to May 2022. They were randomly assigned to either NME plus regular physical therapy (experimental group) or regular physical therapy with strengthening exercises (control group). Assessment was performed at baseline and 8 weeks after the intervention (Fig. 1). This study was conducted in accordance with the Declaration of Helsinki. All individuals were informed about the study and signed an informed consent form before the study. And this study was approved by the ethics committee of the regional hospital and registered with ChiCTR.org.cn (www.chictr.org.cn, 17/12/2021, ChiCTR2100054453).

### Randomization and blinding

Eligible participants were randomly assigned to the experimental or control group by a blinded investigator using computer-generated random numbers through concealed opaque envelopes. An independent blinded therapist assessed participants and collected data at baseline and 8 weeks after treatment. After the baseline examination, another blinded therapist opened the envelope and started the therapy according to the group assignment.

### Participants

Forty participants who have been diagnosed with idiopathic frozen shoulder were recruited from a regional hospital for this study. The inclusion criteria include: (1) FS (adhesive capsulitis) diagnosed at the freezing or frozen stage. (2) symptoms such as shoulder pain, stiffness and joint mobility limitations (limited range of motion of the shoulder in abduction, flexion, extension, internal or external rotation). (3) age over 40 years. (4) unilateral idiopathic FS [28]. Exclusion criteria include: (1) shoulder trauma (rotator cuff injury, etc.). (2) co-occurrence of other neurological or mental disorders (severe cardiac, pulmonary or renal dysfunction, etc.). (3) a previous shoulder surgery. (4) in additional treatments and medications. (5) contraindications to joint mobilization (e.g. osteoporosis). (6) rheumatic diseases (e.g. rheumatoid arthritis). (7) in connection with systemic diseases such as diabetes mellitus and thyroid diseases [29, 30]. Restriction of passive external rotation in the affected shoulder by less than 50% compared to the opposite shoulder was not considered excluded in this study [7].

### Interventions

Participants in both the experimental group (EG) and the control group (CG) performed a 5-minute warm-up exercise in the form of wall climbing at the beginning and received 40 minutes of regular physical therapy. Participants from CG performed 20 minutes of strengthening exercises. Participants in EG performed NME for 20 minutes.

### Regular physical therapy

Regular physical therapy included Maitland mobilization techniques, stretching exercises and active range of motion exercises [15]. Maitland mobilization techniques [30, 31] consisted of distraction of the glenohumeral joint, glenohumeral caudal gliding movements, glenohumeral posterior-anterior gliding movements and glenohumeral anterior-posterior gliding movements. The oscillatory movements were performed with 2–3 gliding movements/second, 30 seconds/set and 5 sets for each gliding movement. The degree of Maitland mobilization depended on the stiffness and pain tolerance of the individual. Shoulder stretching exercises [15] were performed in a standing position using a wand for flexion, extension, abduction, internal rotation and external rotation. Ten seconds/set, 20 sets for each direction. Five seconds rest between two sets. AROM exercises [15] were performed in a standing position for flexion, extension and abduction and in a lying position for internal rotation and external rotation. Ten repetitions/set, 3 sets for each direction. Five seconds rest between two sets.

### Strengthening exercises

Strengthening exercises included isometric and isotonic exercises. Theraband isometric exercises and 1–2-kg dumbbells isotonic exercises were performed for flexion, extension, abduction, internal rotation and external rotation in a standing and lying position. Ten seconds/set, 10 sets for each direction for isometric exercises. 10 repetitions/set, 3 sets for each direction for isotonic exercises. 5 seconds rest between two sets [13].

### NME

**Fig. 2:**
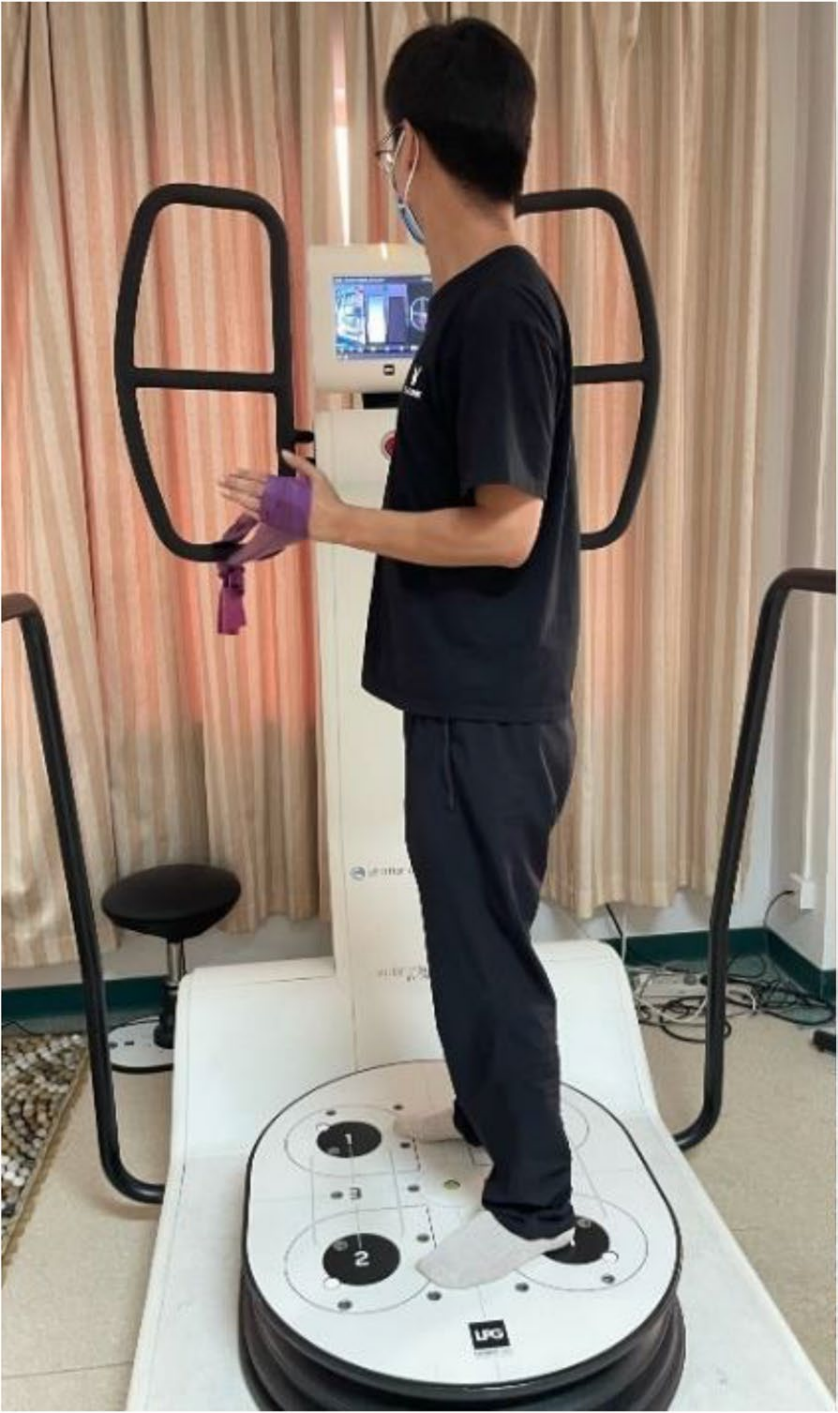
NME for the left shoulder in external rotation

**Fig. 3:**
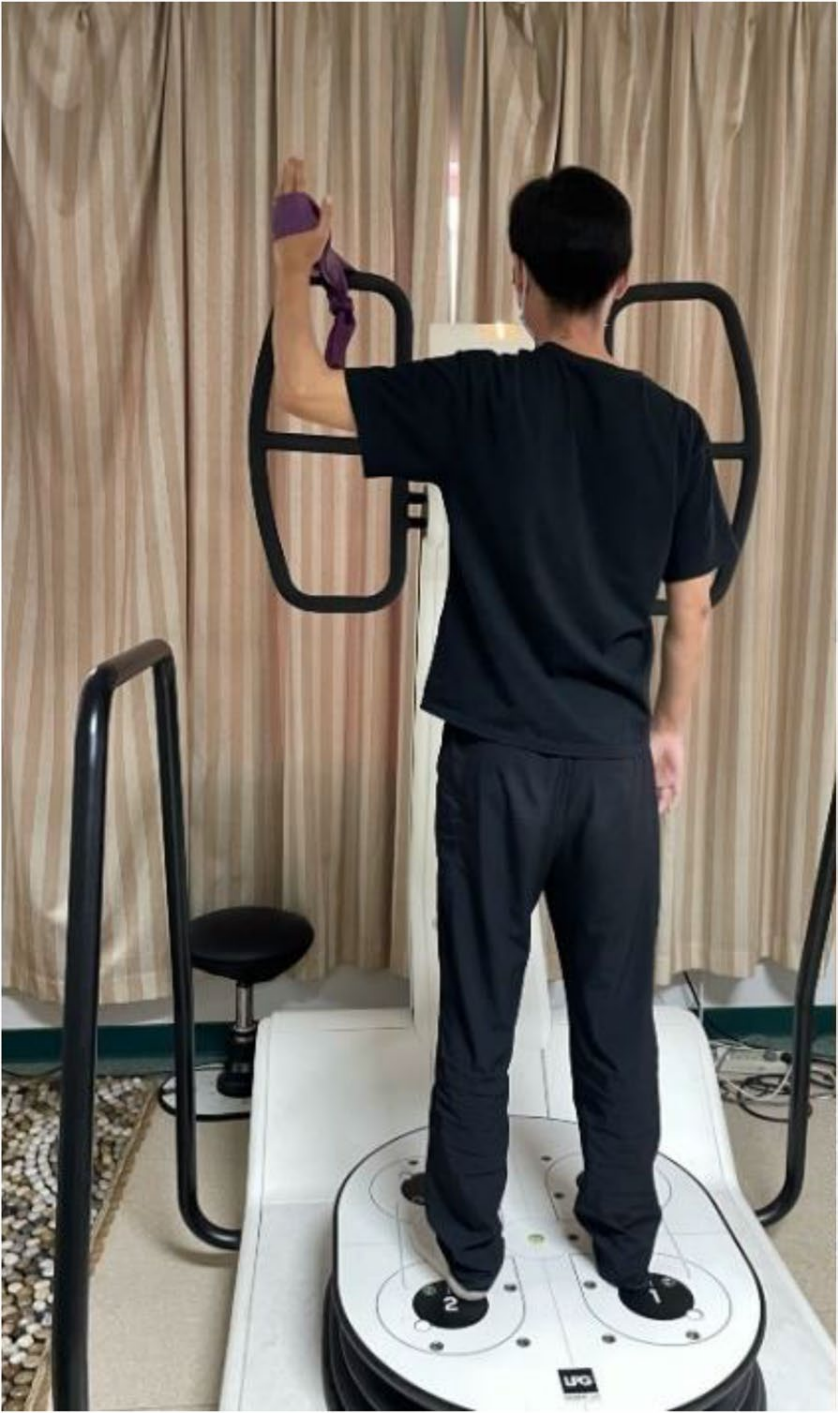
NME for the left shoulder in abduction 90° and external rotation

**Fig. 4:**
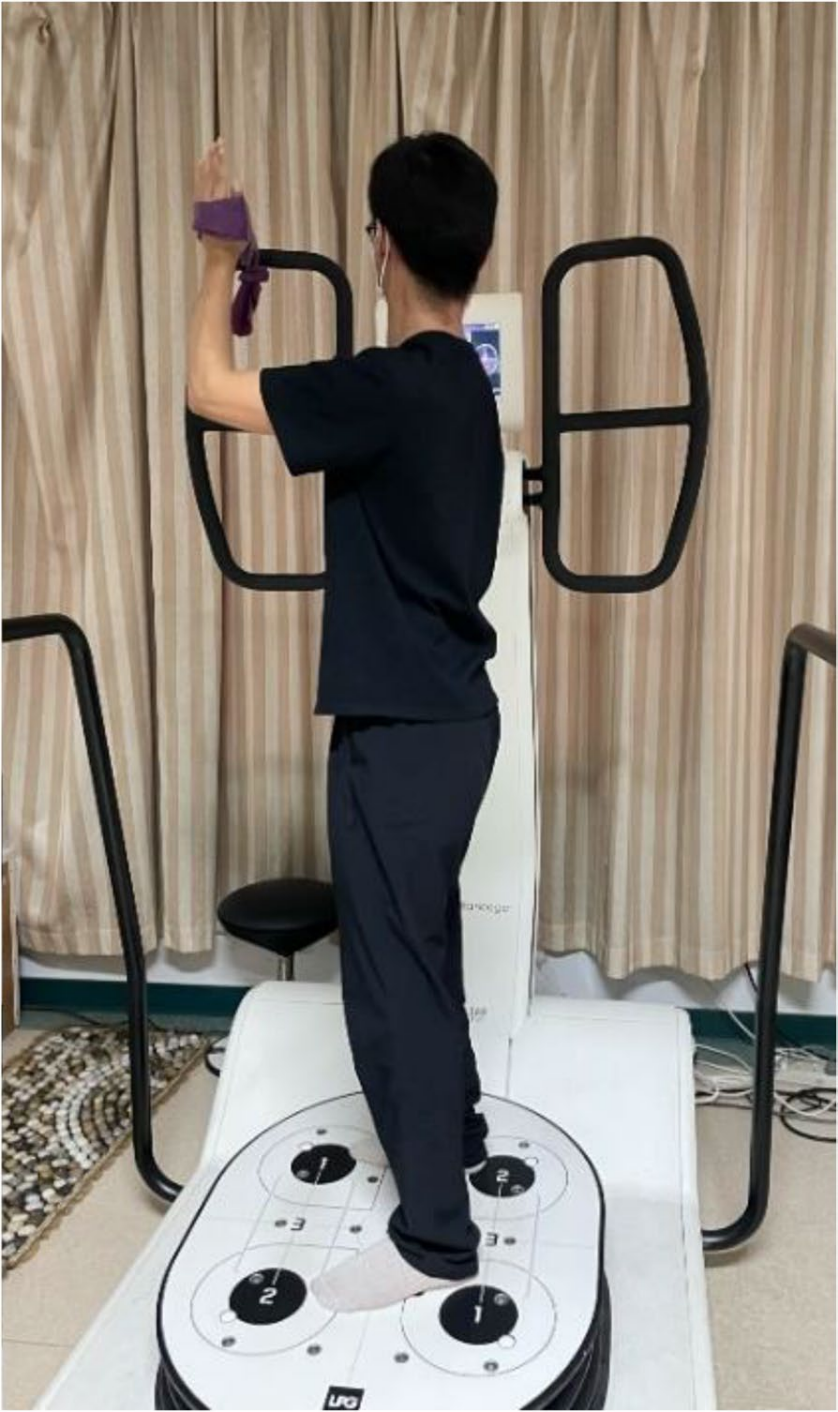
NME for the left shoulder in flexion 90° and external rotation

NME used the HUBER360^a^ in this study, integrating strength, coordination, balance and proprioception exercise [21]. The device provides a multi-axis motorized rotating platform, which can capture a variety of different speeds, amplitude, acceleration trajectory, and real-time monitoring of the individual’s body center of gravity, and provide visual feedback [32]. Individuals tried their best to keep the center of gravity within the target zone through the visual feedback on the screen by holding the elastic belt tied to the armrest when standing on the swaying platform. A stop bottom was controlled by a therapist in case of an emergency. The NME includes six exercises (for the left shoulder, as an example): holding the elastic belt with the shoulder in (1) external rotation (Fig. 2); (2) internal rotation; (3) abduction 90° and external rotation (Fig. 3); (4) abduction 90° and internal rotation; (5) flexion 90° and external rotation (Fig. 4); and (6) flexion 90° and internal rotation (10 seconds of training with 10 seconds rest/repetition × 8 repetitions). There is a one-minute interval for changing directions. Each exercise includes five levels. A score would be shown on the screen according to the exercise performance of the individuals after each session of exercise. If the score was more than 90 points (full score of 100 points), the exercise would go to the next level with higher speed, acceleration, and amplitude of the platform. All treatments are performed and supervised by the same professional, experienced physiotherapist in a quiet environment (once a day, 5 days per week for 8 weeks).

### Outcome assessments

After the treatment and at the beginning of the study, all outcomes were assessed. The same experienced therapist collected data on basic demographic factors such as age, gender, duration of symptoms, stage, affected side and clinical outcomes such as pain and active range of motion (AROM).

Pain intensity was assessed using the visual analogue scale (VAS), which ranks pain from 0 (no pain) to 10 (most severe pain) and has good reliability with an intraclass correlation coefficient (ICC) of 0.71–0.99. The standard error of measurement (SEM) of VAS was 0.03 and the minimum detectable change (MDC) was 0.08 [33, 34].

The shoulder active range of motion (AROM) including flexion (FL), extension (EX), abduction (AB), external rotation (ER), and internal rotation (IR) was measured with a two-arm standard goniometer with a good reliability (ICC 0.91 to 0.99) [35]. The MDC was 6° to 11° and the SEM was 2.4° to 17.1° [36, 37].

For each movement, three measurements were recorded, and the mean was used for statistical analysis. In the standing posture, shoulder flexion and abduction were assessed. In the prone position, shoulder extension was measured with the elbow flexed to 90°. In the prone position, shoulder internal rotation was measured with the shoulder abducted to 90° and the elbow flexed to 90°. Finally, in the supine position, the external rotation of the shoulder was measured with the shoulder abducted to 90° and the elbow flexed to 90° [38].

### Sample size calculation

The sample size calculation was based on the time-by-group interaction of a two-by-two mixed analysis of variance (ANOVA). The effect size was estimated to be 0.25 with 80% power and an α-value of 0.05. G*Power v.3.1.9.2 was used for this calculation. The estimated required sample size was 17 individuals per group. Considering the potential for loss, we aimed to recruit 20 participants per group.

### Statistical analysis

All statistical analyses were performed using SPSS version 19.0^b^. The Kolmogorov-Smirnov test was performed to assess the normal distribution of the data. Continuous variables were presented as means with SDs for normal distribution or median (95% CI) for abnormal distribution. Independent t-tests or Mann-Whitney U test were used to compare demographic data such as age, duration and stage between the two groups. The chi-square test was used to compare differences in gender and affected side between the two groups. Comparisons of VAS and AROM between the experimental and control groups were performed using two-by-two mixed ANOVA with time (pre-intervention and post-intervention) as within-subjects factor and group (NME and control) as between-subjects factor, and effect sizes (η2) were calculated. The main hypothesis of interest was the group × time interaction. Statistical significance was set at 0.05.

## Results

Forty participants (21 males and 19 females) with an average age of 54.23 ± 5.51 years who were diagnosed with idiopathic frozen shoulder were recruited from a regional hospital from December 2021 to May 2022. They were randomly assigned to the experimental or control group and completed the study without adverse effects. The data for age, duration, VAS score and all AROM were normally distributed, the data for stage was not normally distributed. At baseline, there were no differences in demographics between groups as seen in Table 1. The two-by-two mixed ANOVA revealed a significant group-by-time interaction for VAS (F = 29.67; *p* < 0.01; η^2^ = 0.438); AROM in FL (F = 12.05; *p* < 0.01; η^2^ = 0.241), IR (F = 6.62; *p* < 0.05; η^2^ = 0.148), ER (F = 16.93; *p* < 0.01; η^2^ = 0.308). There was a non-significant time-by-group interaction for AB (*p* = 0.05) and EX (*p* > 0.05). The two-by-two mixed ANOVA revealed a significant main effect of time for VAS (F = 1648.47; *p* < 0.01; η^2^ = 0.977); AROM in FL (F = 591.70; *p* < 0.01; η^2^ = 0.940), EX (F = 114.57; *p* < 0.01; η^2^ = 0.751), AB (F = 1602.04; *p* < 0.01; η^2^ = 0.977), IR (F = 664.14; *p* < 0.01; η^2^ = 0.946) and ER (F = 1096.92; *p* < 0.01; η^2^ = 0.967). There was a non-significant main effect of the group for VAS and all AROM as seen in Table 2.

**Table 1:** Demographic characteristics

Characteristics	Experimental group	Control group	P
	(EG) *n* = 20	(CG) *n* = 20	
Age, years	53.60 ± 5.18	54.85 ± 5.89	0.480
Gender,male/female	12/8	9/11	0.342
Duration, months	2.79 ± 1.05	3.13 ± 1.07	0.310
Stage	1.30 (1.08–1.52)	1.15 (0.98–1.32)	0.262
Affected side, R/L	13/7	15/5	0.490

*n* number, *R* Right, *L* Left

**Table 2:** Comparison of VAS and AROM

	Experimental Group		Control Group		P	η^**2**^
	Pre-intervention	Post-intervention	Pre-intervention	Post-intervention		
Pain on VAS	7.05 ± 1.57	2.40 ± 1.43	6.95 ± 1.32	3.40 ± 1.50	< 0.01	0.438
AROM of FL	92.10 ± 25.00	149.75 ± 21.42	89.60 ± 27.42	132.85 ± 21.31	0.001	0.241
AROM of EX	31.15 ± 7.29	42.05 ± 2.54	32.45 ± 7.98	41.00 ± 4.01	0.204	0.042
AROM of AB	93.80 ± 12.59	156.65 ± 14.62	89.15 ± 11.05	145.95 ± 15.46	0.05	0.097
AROM of ER	22.80 ± 6.61	72.80 ± 14.34	23.90 ± 8.96	62.85 ± 15.05	< 0.01	0.308
AROM of IR	31.65 ± 9.35	69.10 ± 13.93	29.40 ± 9.02	60.05 ± 17.43	0.014	0.148

## Discussion

This study aims to investigate the effects of NME compared to strengthening exercises on pain intensity and AROM in individuals with FS. Significant improvements in VAS, AROM of flexion, internal rotation, and external rotation in the experimental group have been observed in this study.

### Evaluation of the effect of neuromuscular exercise on pain

The results show that pain intensity improved significantly in individuals with FS after an intervention of 40 sessions of NME plus regular physical therapy. These results are consistent with the results of other studies [21, 23, 24] indicating that shoulder pain can be alleviated after NME. Eshoj et al. [21] demonstrated that NME was superior to standard exercises in terms of pain reduction in individuals with anterior shoulder dislocation. Juul-Kristensen et al. [23] proved that shoulder NME was an effective pain treatment in individuals with subacromial pain syndrome. Ager et al. [24] reported a positive effect of upper extremity NME on pain in rotator cuff tendinopathy. Ginn et al. [25] demonstrated that exercises to restore neuromuscular control were as effective as corticosteroid injections in treating shoulder pain in the short term. In addition to the shoulder, NME has a significant therapeutic effect on many other musculoskeletal pain conditions, such as low back pain [39, 40], neck pain [41], chronic musculoskeletal pain in the elderly [42], chronic pain after primary total knee arthroplasty [43], and knee and hip osteoarthritis [44, 45]. NME includes active exercises of strength, coordination, balance, and proprioception. Multimodal active exercises have been proposed for affected individuals to participate in, which may contribute to pain management by activating endogenous pain-inhibitory mechanisms and reducing sensitivity to noxious stimuli, which has been termed “exercise-induced hypoalgesia” [46–48]. Individuals with FS can be prescribed NME as a pain reliever.

### Evaluation of the effect of neuromuscular exercise on AROM

A significant difference was found in the AROM of flexion, internal rotation, and external rotation between the group with NME plus regular physical therapy and the group with regular physical therapy plus strengthening exercises. Shoulder AROM improved after 5 weeks of neuromuscular control exercises in subjects with chronic shoulder pain, as reported by Ginn et al. [25]. Fernandez et al. [49] showed that neuromuscular warm-up exercises in young tennis players resulted in significant improvement in internal and external rotation of the shoulder passive range of motion (PROM). The ability to produce regulated movements through coordinated muscle activity is referred to as neuromuscular control. NME has effects on muscle activation patterns and biomechanics of the surrounding joint musculature [50]. The strength and coordination of muscle exercises involved in NME may be the reason for the increase in AROM, as the shoulder complex relies on muscles to provide dynamic stability and flexibility for AROM [51]. However, evidence for NME is lacking at ROM in FS, and no previous study was found. RCTs of sufficiently good quality are needed to investigate NME in individuals with FS.

### Limitations of the study

First, the sample size in this study was relatively small. Second, it is unclear whether the frequency, intensity, and duration of NME by the motorized device were the best in this study, and there is no previous study to compare it with. Third, only the VAS score and AROM of the shoulder were measured in this study, and no disability questionnaires were reported. Finally, no long-term effect of NME was observed at FS. Therefore, further studies with a larger sample and different frequencies, intensities, and durations on long-term and functional effects are desirable.

## Conclusions

This study shows that NME performed with a motorized device in combination with regular physical therapy in the form of joint mobilization, stretching, and AROM at FS is more effective for pain relief and improvement of AROM compared with regular physical therapy with strengthening exercises. Considering the positive effects on FS, it is recommended that NME could be used in the treatment of individuals with FS. However, the long-term and functional effects of NME on frozen shoulder need to be investigated in future large-scale studies.

### Suppliers

- Huber360 MD; LPG Systems.
- SPSS; IBM Corp.

## Supplementary Information

**Additional file 1.** Intervention detail.

## Data Availability

The datasets used and analyzed during the current study are available from the corresponding author on reasonable request.

## Abbreviations

FS: Frozen Shoulder
NME: Neuromuscular Exercise
RCT: Randomized Controlled Trial
ROM: Range of Motion
PROM: Passive Range of Motion
AROM: Active Range of Motion
EG: Experimental Group
CG: Control Group
VAS: Visual Analogue Scale
SEM: Standard error of measurement
MDC: Minimal detectable change
Fl: Flexion
EX: Extension
AB: Abduction
ER: External Rotation
IR: Internal Rotation
ANOVA: Analysis of variance
