# Supplementary material for: Positive effects of neuromuscular exercises on pain and active range of motion in idiopathic frozen shoulder: a randomized controlled trial
Source: BMC Musculoskelet Disord. 2023 Jan 20;24:50. doi: 10.1186/s12891-023-06173-8 (PMC9854051; doi:10.1186/s12891-023-06173-8)
Supplement: Supplementary file 1 — Additional file 1. Intervention detail. [file 12891_2023_6173_MOESM1_ESM.docx]

Additional file 1 Rehabilitation for frozen shoulder

| Intervention | Description |
| --- | --- |
| Maitland’s mobilization  Stretching exercise  AROM exercise | Distraction of glenohumeral joint, glenohumeral caudal glides, glenohumeral posterior-anterior glides, and glenohumeral anterior-posterior glides. The oscillatory movements were performed at 2-3 glides/second, 30 seconds/set, and 5 sets for each glide. The grades of Maitland mobilization depended on the subject’s stiffness and tolerance of pain.  Shoulder stretching exercises for flexion, extension, abduction, internal rotation and external rotation in a standing position using a wand. 10 seconds/set, 20 sets for each direction. 5 seconds rest between two sets.  Active range of motion exercises for flexion, extension and abduction in a standing position, internal rotation and external rotation in a lying position. 10 repetitions/set, 3 sets for each direction. 5 seconds rest between two sets. |
| Strengthening exercise | Theraband isometric exercises and 1-2-kg dumbbells isotonic exercises for flexion, extension, abduction, internal rotation and external rotation in a standing and lying position. 10 seconds/set, 10 sets for each direction for isometric exercises. 10 repetitions/set, 3 sets for each direction for isotonic exercises. 5 seconds rest between two sets. |
| NME | Using the HUBER360 system, participants tried their best to keep the center of gravity within the target zone through the visual feedback on the screen by holding the elastic belt tied to the armrest when standing on the swaying platform. Participants held the elastic belt with the shoulder in (1) external rotation; (2) internal rotation; (3) abduction 90° and external rotation; (4) abduction 90° and internal rotation; (5) flexion 90° and external rotation; and (6) flexion 90° and internal rotation. 10- second training with 10-second rest/set, 8 sets for each exercise. There is a one-minute interval for changing directions. |


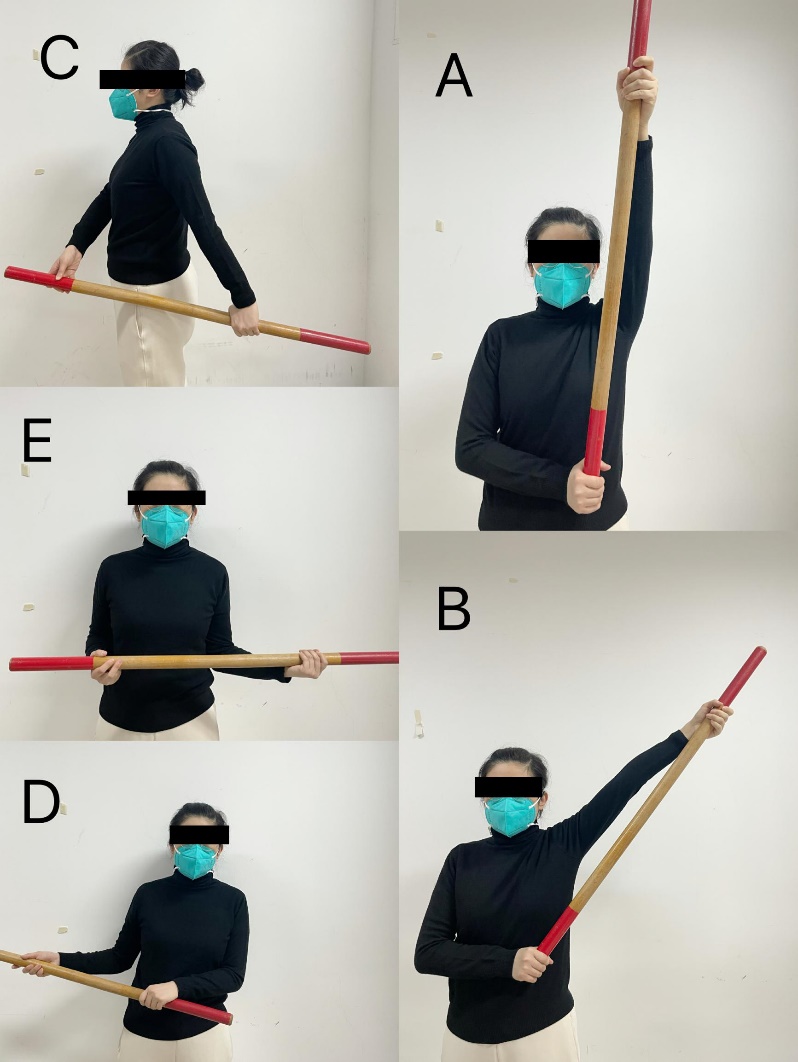


Shoulder stretching exercise (left side): flexion (A), abduction (B), extension (C),

internal rotation (D), external rotation (E).


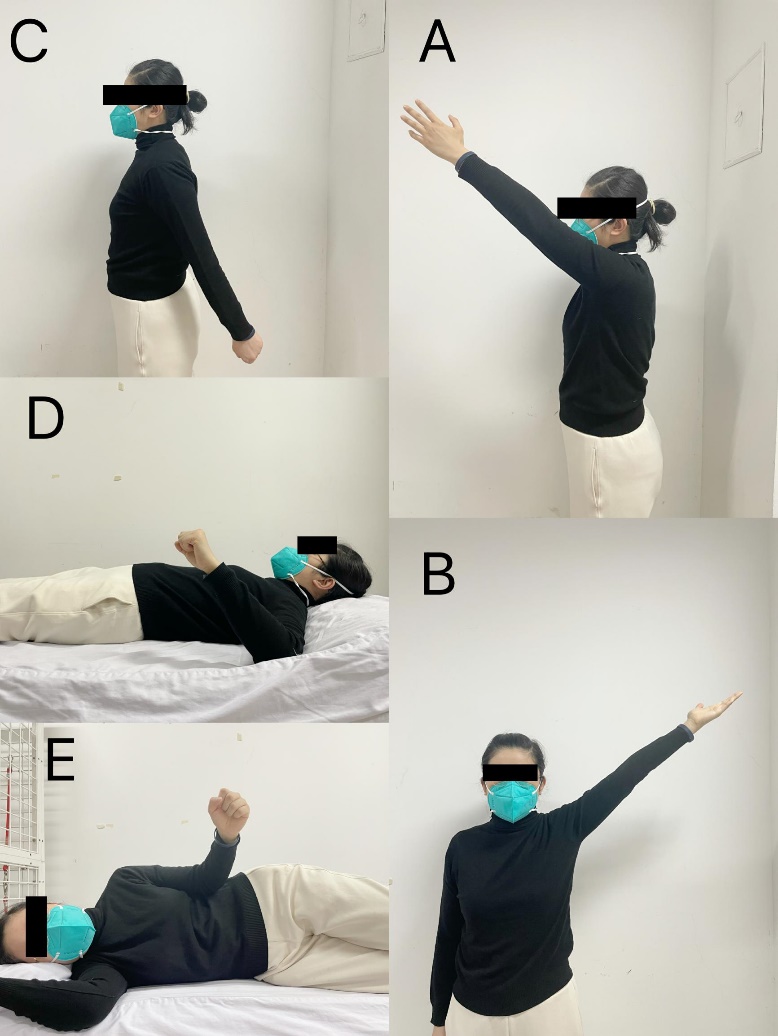


Shoulder AROM exercise (left side): flexion (A), abduction (B), extension (C),

internal rotation (D), external rotation (E).


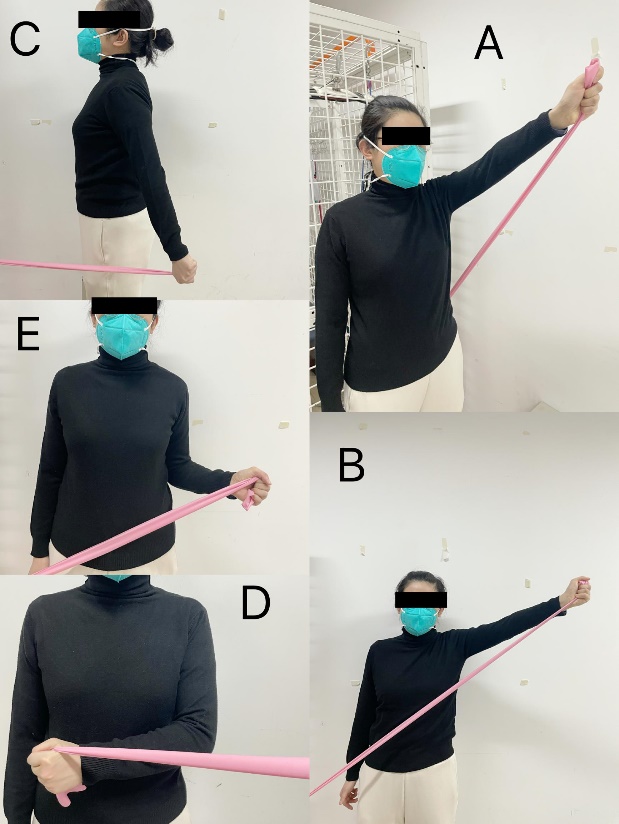


Shoulder strengthening exercise using a Theraband (left side): flexion (A), abduction (B), extension (C), internal rotation (D), external rotation (E).


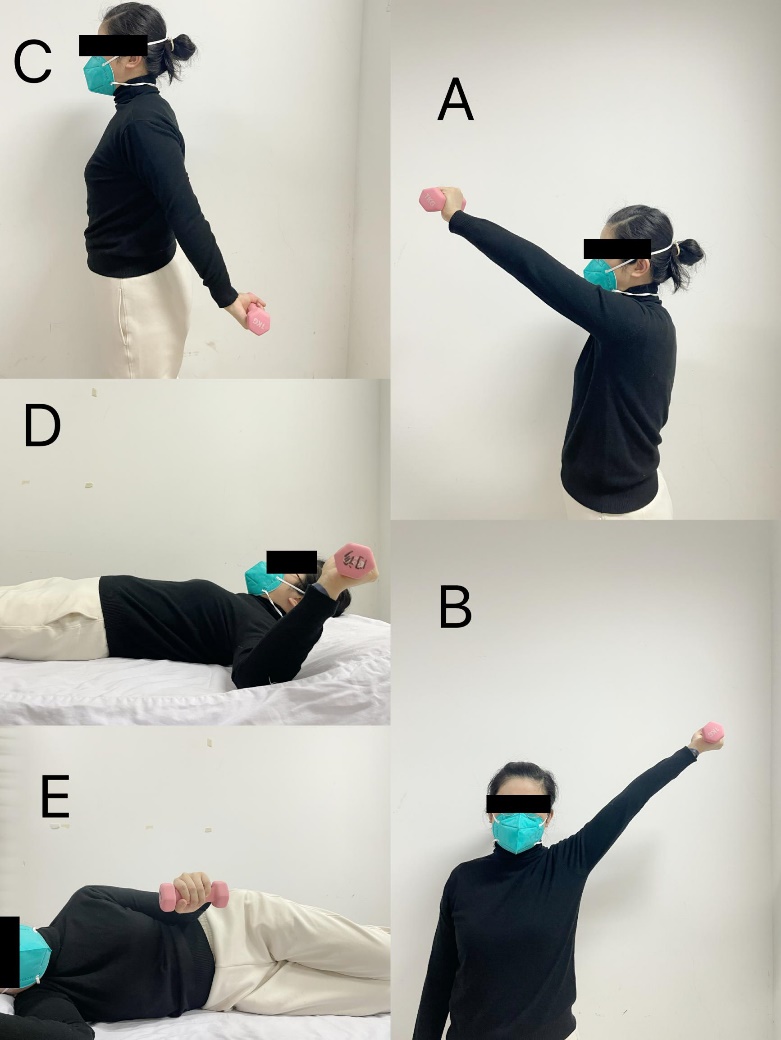


Shoulder strengthening exercise using a dumbbell (left side): flexion (A), abduction (B), extension (C), internal rotation (D), external rotation (E).
